# Integrative Bioinformatics Links HNF1B with Clear Cell Carcinoma and Tumor-Associated Thrombosis

**DOI:** 10.1371/journal.pone.0074562

**Published:** 2013-09-09

**Authors:** Justin Cuff, Keyan Salari, Nicole Clarke, Ghada E. Esheba, Andrew D. Forster, Stephanie Huang, Robert B. West, John P. Higgins, Teri A. Longacre, Jonathan R. Pollack

**Affiliations:** 1 Department of Pathology, Stanford University, Stanford, California, United States of America; 2 Department of Genetics, Stanford University, Stanford, California, United States of America; 3 Department of Pathology, Faculty of Medicine, Tanta University, Tanta, Egypt; The University of Hong Kong, China

## Abstract

Clear cell carcinoma (CCC) is a histologically distinct carcinoma subtype that arises in several organ systems and is marked by cytoplasmic clearing, attributed to abundant intracellular glycogen. Previously, transcription factor hepatocyte nuclear factor 1-beta (HNF1B) was identified as a biomarker of ovarian CCC. Here, we set out to explore more broadly the relation between HNF1B and carcinomas with clear cell histology. HNF1B expression, evaluated by immunohistochemistry, was significantly associated with clear cell histology across diverse gynecologic and renal carcinomas (*P*<0.001), as was hypomethylation of the *HNF1B* promoter (*P*<0.001). From microarray analysis, an empirically-derived HNF1B signature was significantly enriched for computationally-predicted targets (with HNF1 binding sites) (*P*<0.03), as well as genes associated with glycogen metabolism, including glucose-6-phophatase, and strikingly the blood clotting cascade, including fibrinogen, prothrombin and factor XIII. Enrichment of the clotting cascade was also evident in microarray data from ovarian CCC *versus* other histotypes (*P*<0.01), and HNF1B-associated prothrombin expression was verified by immunohistochemistry (*P* = 0.015). Finally, among gynecologic carcinomas with cytoplasmic clearing, HNF1B immunostaining was linked to a 3.0-fold increased risk of clinically-significant venous thrombosis (*P* = 0.043), and with a 2.3-fold increased risk (*P* = 0.011) in a combined gynecologic and renal carcinoma cohort. Our results define HNF1B as a broad marker of clear cell phenotype, and support a mechanistic link to glycogen accumulation and thrombosis, possibly reflecting (for gynecologic CCC) derivation from secretory endometrium. Our findings also implicate a novel mechanism of tumor-associated thrombosis (a major cause of cancer mortality), based on the direct production of clotting factors by cancer cells.

## Introduction

Ovarian clear cell carcinoma (CCC) represents 5–10% of ovarian cancers and is distinguished by a poor response to combination (including platinum-based) chemotherapy, unfavorable prognosis when presenting at high stage, and venous thromboembolism [1]. The clear cell change itself is a light microscopic finding defined by pale or clear cytoplasm which is attributed to an accumulation of glycogen. While cytoplasmic clearing has been described in many different tumor types, this phenotype is most frequently manifested in renal and ovarian tumors. In fact, the morphologic resemblance between renal and ovarian clear cell carcinomas led to the initial designation of ovarian clear cell carcinoma as mesonephric carcinoma [2].

Gene-expression profiling of ovarian carcinomas identified HNF1B (hepatocyte nuclear factor 1-beta) to be amongst the most upregulated transcripts in ovarian CCC (compared to other histotypes) [3], [4]. Initial immunohistochemical (IHC) characterization of HNF1B expression confirmed selective expression in the ovarian CCC histotype [4]–[6], and suggested utility as a tissue biomarker. In subsequent studies, the *HNF1B* promoter was found hypomethylated in ovarian CCC *vs*. other histotypes [7], [8]. Further, RNA interference studies in ovarian CCC cell lines revealed a dependency on HNF1B, where knockdown led to apoptotic cell death, nominating HNF1B as a possible target for therapy [4].

HNF1B (also known as TCF2) is a member of the homeodomain-containing transcription factors, a superfamily with diverse roles in development and often deregulated in cancer [9]. HNF1B itself was originally characterized as a hepatic transcription regulator, where it can also heterodimerize with the related HNF1A (both recognize the same DNA binding site) [10]. HNF1B has since been found expressed more widely, including in the developing and adult liver, kidney, pancreas, lung and intestine [11]–[13].

Mutations in *HNF1B* are associated with a spectrum of human diseases. Mutations were first reported in a subset of patients with maturity onset diabetes of the young (MODY5) [14], an autosomal-dominant, early-onset form of type 2 diabetes. HNF1B has since been linked to structural abnormalities and dysfunction of the kidney (most often renal cysts), pancreas, liver and genital tract [15], [16]. These mutations, along with analogous phenotypes in mice [17], underscore the key roles of HNF1B in the development and function of these respective organs.

More recently, genome-wide association studies have linked DNA sequence variants within the second intron of *HNF1B* to both an increased risk of prostate cancer, and a protective effect against type 2 diabetes [18], [19]. These reports further define the pleiotropic roles of HNF1B in human health and disease.

The association of HNF1B expression with ovarian cancer clear cell change (noted by glycogen accumulation), along with its connection to glucose homeostasis, led us to investigate a broader relation between HNF1B (and its transcriptional network) and cytoplasmic clearing. Here, by IHC and integrative computational analysis, we identify HNF1B as a marker of cytoplasmic clearing across diverse tumor types, supporting a likely direct role in glycogen accumulation. We also uncover a surprising link to blood clotting factors, with important implications for understanding and possibly managing the hypercoagulable state associated with clear cell malignancy.

## Materials and Methods

### Specimens

Formalin-fixed paraffin-embedded and freshly-frozen tissue specimens were obtained from the Stanford Department of Pathology archives. These existing specimens and associated clinical data were used with the approval of the Stanford University Institutional Review Board (IRB), with waiver of patient consent based on OHRP 45 CFR 46.116(d): minimal risk, no adverse affects to subjects’ rights/welfare, and practicality. Associated venous thromboembolic events were identified by review of patient medical records (where available), accessed via the Stanford Translational Research Integrated Database Environment (STRIDE) [20]. Criteria for tumor-associated thromboembolism were: (i) Any patient with clinical documentation of a thromboembolic event that was not explained by an alternative medical condition (e.g. atrial fibrillation or carotid atherosclerosis in the case of stroke patients, and Factor V Leiden or a lupus anti-coagulant in patients with DVT); and (ii) The thromboembolic event had to have occurred either within the two years preceding the cancer diagnosis, or if after the diagnosis must have been associated with a recurrence of the tumor. Additional freshly-frozen specimens (for Q-RT-PCR analysis) were obtained from the Stanford Tissue Bank, with IRB approval and patient consent.

### Immunohistochemistry

HNF1B expression was assessed by IHC, using a monoclonal antibody directed against HNF1B (clone C-20, Santa Cruz Biotechnology, titer 1∶2,000). An anatomically and histologically diverse set (n = 1,493) of tissue microarray and conventional tissue sections enriched for gynecologic (n = 85) and renal (n = 295) primaries with cytoplasmic clearing was evaluated. Nuclear localization was required for scoring, and the intensity and extent of expression was recorded as either: negative or positive (either focal/weak expression or diffuse and strong). Prothrombin expression was evaluated using a monoclonal antibody (clone 095, Enzyme Research Laboratories, titer 1∶500) [21], and cytoplasmic staining was recorded as negative (0% of cells staining), weak (1–5%), moderate (5–50%) or strong (>50%). Statistical analyses were done using the Fisher’s exact test, with significance ascribed to *P* values<0.05.

### DNA Methylation Analysis

Promoter methylation of *HNF1B* was evaluated by bisulfite sequencing. Genomic DNA was prepared from macrodissected freshly-frozen specimens using the DNeasy Blood & Tissue kit (Qiagen). Bisulfite modification was carried out using the EZ DNA methylation kit (Zymo Research) according to the manufacturer’s protocol. A 191 bp region within the CpG island of HNF1B exon 1 was PCR-amplified using previously published [8] primers, 5′-GGGGTYGAGTTYGATATTAAGT-3′ (forward) and 5′-TACCTAAACATCCRATCCACCT-3′ (reverse), designed to amplify both methylated and unmethylated bisulfite-modified DNAs. PCR products were then purified by agarose gel electrophoresis and analyzed by Sanger dideoxy DNA sequencing.

### Bioinformatic Analysis

A computationally predicted set of HNF1B transcriptional targets (n = 259), based on the V$HNF1_Q6 TRANSFAC [22] binding site matrix (i.e. containing the motif WRGTTAATNATTAACNNN within promoter regions [−2 kb to +2 kb] around the transcription start site), was obtained from the Molecular Signatures Database [23]. Gene set enrichment analysis (GSEA) was used to evaluate enrichment of predicted HNF1B targets among the genes upregulated by tetracycline-induced expression of HNF1B in HEK293 embryonic kidney cells, using a publicly-available dataset (GEO repository accession GSE3308) [24]. Default GSEA parameters were used, except that gene sets were permuted rather than phenotypes (because of the small sample number). GSEA (with default parameters) was also used to evaluate enrichment of computationally validated HNF1B targets among genes relatively overexpressed in ovarian carcinoma with clear cell *vs*. other histotypes, using a publicly-available dataset (GSE6008) [25]. Overlaps between leading edge genes and functional (curated) gene sets (n = 1,892) were evaluated using the Molecular Signatures Database “compute overlaps” function. Expression levels of select clotting factor genes were also evaluated in a public microarray dataset of laser microdissected ovarian CCC *vs*. normal ovarian surface epithelium (GSE29450) [26].

### Quantitative-RT-PCR

Transcript levels of select clotting factor genes were assayed in an independent sample set by Q-RT-PCR. RNA was prepared from macrodissected freshly-frozen specimens using the RNeasy Mini kit (Qiagen). Reverse transcription of RNA was done using SuperScript III (Invitrogen) according to the manufacturer. Q-RT-PCR was then carried out using TaqMan Gene Expression assays (ABI) and TaqMan Fast Universal PCR Master Mix kit (ABI), on an ABI 7500 sequence detection system as per manufacturer’s instructions. We used the ΔCT method to calculate relative clotting factor transcript levels (FAM-labeled probes) normalized to internal reference GAPDH (VIC-labeled probe), which was then expressed as a ratio to the Ct value of a single ovarian serous carcinoma “reference” sample (also normalized to GAPDH). Q-RT-PCR assay primers (ABI) were as follows: HNF1B (Hs01001602_m1); F2 (Hs01011988_m1); FGA (Hs00241027_m1); FGB (Hs00905942_m1); GAPDH (Hs02758991_g1).

## Results

### HNF1B Expression is Broadly Associated with Cytoplasmic Clearing

Previously by microarray analysis, HNF1B was discovered to be upregulated in ovarian CCC as compared to other histotypes [3], [4], a finding confirmed by IHC [4]–[6]. To further evaluate HNF1B as a biomarker in ovarian CCC, and to more broadly characterize its relation to cytoplasmic clearing, we carried out IHC on tissue microarray sections representing anatomically and histologically diverse tumors, enriched for renal and gynecologic primaries with cytoplasmic clearing (Table S1).

**Figure 1:**
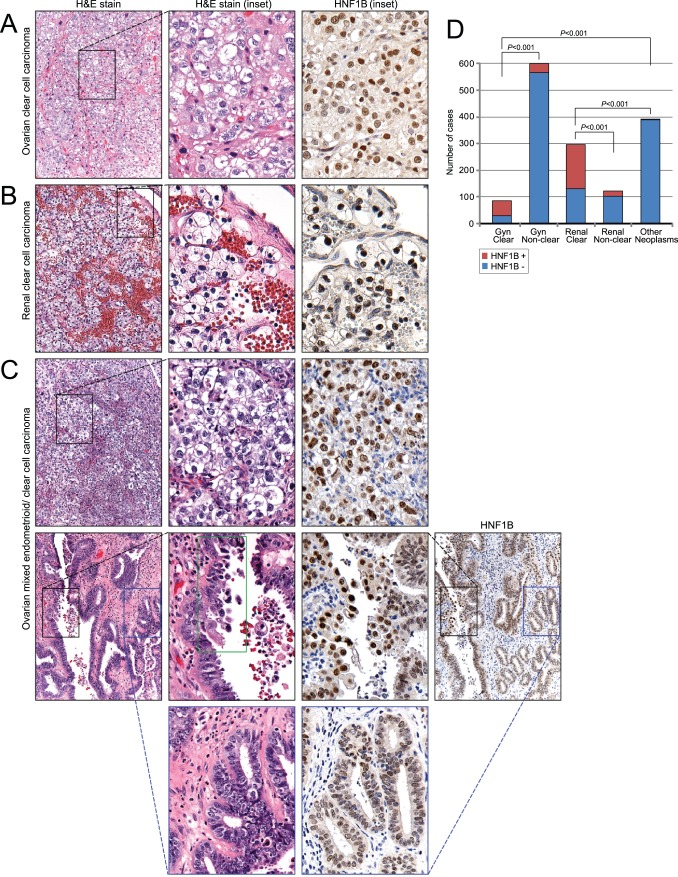
Nuclear HNF1B expression is associated with cytoplasmic clearing across multiple tumor types. Representative H&E (10×objective, and magnified inset) and positive HNF1B immunostains are shown for (**A**) ovarian CCC, (**B**) renal CCC, and (**C**) mixed endometrioid/clear cell ovarian carcinoma. In the ovarian carcinoma with mixed histology (C), the upper row of panels depicts a region of the tumor with clear cell histology (HNF1B-positive); the middle row of panels (insets) depicts a different region of the same tumor with endometrioid histology and clear cell features (note papillary pattern, highlighted by green box) (also HNF1B-positive); and the bottom row (insets) depicts yet another region of the same tumor with endometrioid histology but without clear cell features (and with correspondingly weaker HNF1B-immunostaining). Note that this patient experienced a tumor-associated thromboembolic event (see main text). (**D**) Graphical display of proportion of HNF1B positive cases (shaded red) among different carcinoma types with or without cytoplasmic clearing. Gynecologic carcinomas include those from the endometrium, cervix and ovary. Other neoplasms represent diverse anatomic sites (see Table S1). *P*-values (two-sided Fisher’s exact test) for pairwise comparisons are indicated.

As expected, positive immunostaining of this transcription factor was associated with predominantly nuclear expression (Fig. 1A–C). However, not only did HNF1B stain ovarian CCC, but expression was also detected in other tumors with cytoplasmic clearing, including renal CCC, endometrial carcinoma, germ cell tumors with yolk sac elements, and ovarian and endometrial carcinomas with mixed histology. In total, we observed HNF1B expression in 66% of gynecologic and 56% of renal neoplasms with cytoplasmic clearing, compared to 5% and 16%, respectively, without cytoplasmic clearing, and compared to 1% of other neoplasms, all highly significant differences (*P*<0.001; two-tailed Fisher exact tests) (Fig. 1D, and Table S1). This finding suggests a possible general role of HNF1B in cellular processes leading to cytoplasmic clearing. The only tumors without cytoplasmic clearing that were found to express HNF1B were renal papillary carcinomas, some endometrioid carcinomas, and uncommonly gastrointestinal carcinomas (Table S1).

### HNF1B Promoter Hypomethylation is Associated with Cytoplasmic Clearing

**Figure 2:**
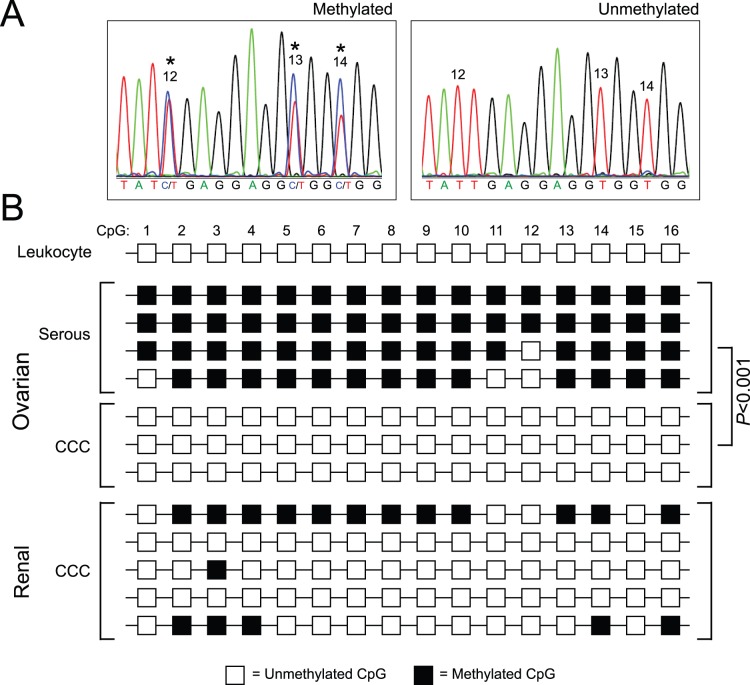
*HNF1B* CpG island is hypomethylated in carcinomas with cytoplasmic clearing. (**A**) Representative bisulfite-modified DNA sequences indicative of CpG island methylation (*left*) and hypomethylation (*right*). CpG methylation (at numbered sites) is indicated with an asterisk. (**B**) Schematic summary of CpG methylation within a 148bp region (PCR primers excluded) of the CpG island spanning *HNF1B* exon 1. The numbered boxes represent each of the 16 CpG dinucleotides ordered across the region; black fill denotes presence of methylation. Data are shown for individual specimens, including normal blood leukocytes, ovarian carcinomas of serous (n = 4) and clear cell (n = 4) histotype, and renal CCC (n = 5). The proportion of methylated CpGs is significantly higher among serous *vs*. clear cell ovarian cancers (*P*<0.001; Student’s t-test).

Epigenetic marks, including DNA methylation, control gene expression and are commonly altered in cancer [27]. Promoter hypomethylation of *HNF1B*, consonant with active transcription, was previously reported in ovarian CCC [7], [8]. To investigate the relation between promoter methylation, HNF1B expression, and cytoplasmic clearing more broadly, we characterized HNF1B promoter methylation in freshly-frozen ovarian cancers of serous and clear cell histotype, as well as in renal CCC. A fragment of the CpG-rich region (CpG island) overlapping exon 1 of *HNF1B*, and harboring 16 scorable CpG dinucleotides, was evaluated by bisulfite sequencing. In each of three ovarian CCC cases, no DNA methylation was detected (Fig. 2). In contrast, in each of four ovarian serous carcinoma cases, methylation was detected at most or all of the 16 CpG sites, a highly significant difference (*P*<0.001; Student’s t-test) (Fig. 2B). In all instances, the observed CpG methylation was partial (Fig. 2A), possibly reflecting hemizygous methylation, tumor heterogeneity, or more likely intermixed stromal “contamination”.

Among five renal CCC cases, all but one exhibited a hypomethylation pattern more comparable to ovarian CCC (Fig. 2B). The freshly-frozen specimens were expended for DNA isolation, precluding a direct assessment of the relation between HNF1B promoter methylation and gene expression. Nonetheless, in combination with the above IHC results, our findings suggest that promoter hypomethylation is permissive (though likely not sufficient) for HNF1B expression in both ovarian and renal carcinomas with cytoplasmic clearing.

### HNF1B Targets Relate to Starch Metabolism and the Clotting Cascade

**Figure 3:**
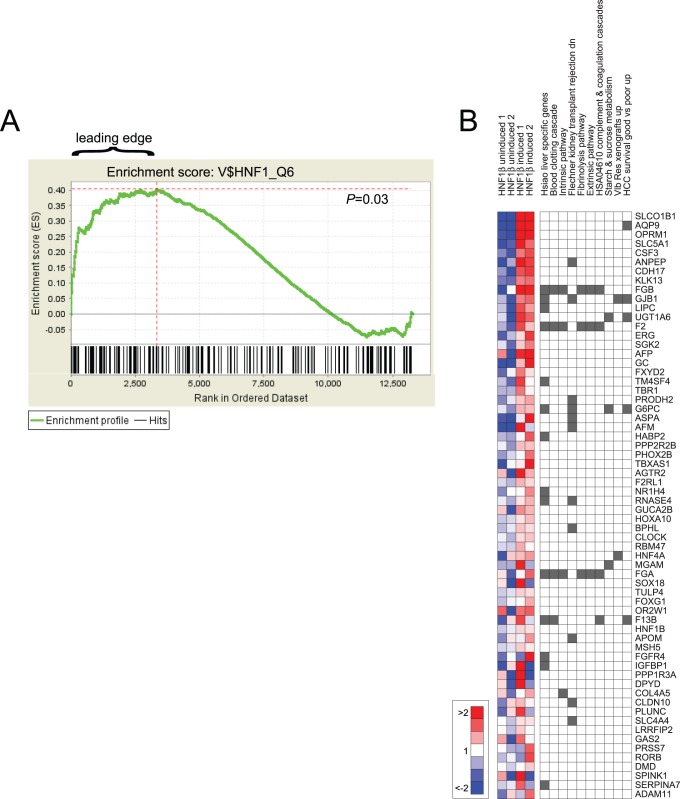
HNF1B transcriptional targets are enriched for starch metabolism and clotting cascade pathways. (**A**) GSEA analysis shows enrichment of computationally predicted (by binding sites) HNF1 transcriptional targets (n = 259) among genes upregulated following tetracycline-induced HNF1B expression in HEK293 kidney cells [24]. (**B**) The “leading edge” enriched genes (n = 64) are significantly over-represented among select functional gene sets (nominal *P* values<0.01); the top 10 gene sets are shown. Columns on the left depict relative log_2_ gene expression levels (red/blue scale indicated) among replicate cell lines with or without HNF1B induction. Columns on the right indicate membership (gray fill) among the top 10 most significant gene sets, which include liver-specific genes, clotting cascade (represented by five different gene sets), and starch/sucrose metabolism.

To explore the connection between HNF1B and clear cell change, we applied an integrative bioinformatics approach to identity putative transcriptional targets. First, using a TRANSFAC binding site matrix (V$HNF1_Q6), we identified 259 genes with an HNF1 binding site within 2 Kb of the transcription start site. To assess the validity of these putative targets, we determined the extent to which they were altered upon induction of HNF1B in HEK293 embryonic kidney cells, a biologically-relevant context (given the role of HNF1B in kidney morphogenesis, and its expression in renal CCC), and one for which microarray data were publicly available [24]. By Gene Set Enrichment Analysis (GSEA) [23], the set of HNF1B putative targets was significantly enriched among the genes upregulated by HNF1B (*P*<0.03; Fig. 3A), indicating that many are true biological targets. Comparable results were obtained using other TRANSFAC HNF1 binding site matrices ($VHNF1_C, $VHNF1_01, and RGTTAMWNATT_$VHNF1_01).

The subset of HNF1B putative targets within the “leading edge” of the enrichment profile (i.e. those most upregulated upon HNF1B induction) (Fig. 3A) are likely to be enriched for true biologic targets. To objectively characterize the biological pathways and processes populated by these genes, we computed overlaps between the leading edge (n = 64) and 1,892 curated genes sets (representing chemical and genetic perturbations, and canonical pathways) from the Molecular Signatures Database [23]. The top ten significant overlapping gene sets (nominal *P* values<0.01; false discover rates<25%) (Fig. 3B) included liver specific genes (14 genes), a starch & sucrose metabolism (including glucose-6-phophatase; *G6PC*) gene set, and most remarkably, five different gene sets relating to the blood clotting cascade, where common gene members included fibrinogen (*FGA*, *FGB*), prothrombin (*F2*), and factor XIII (*F13B*).

### Ovarian CCC Express HNF1B Targets Including Clotting Factors

**Figure 4:**
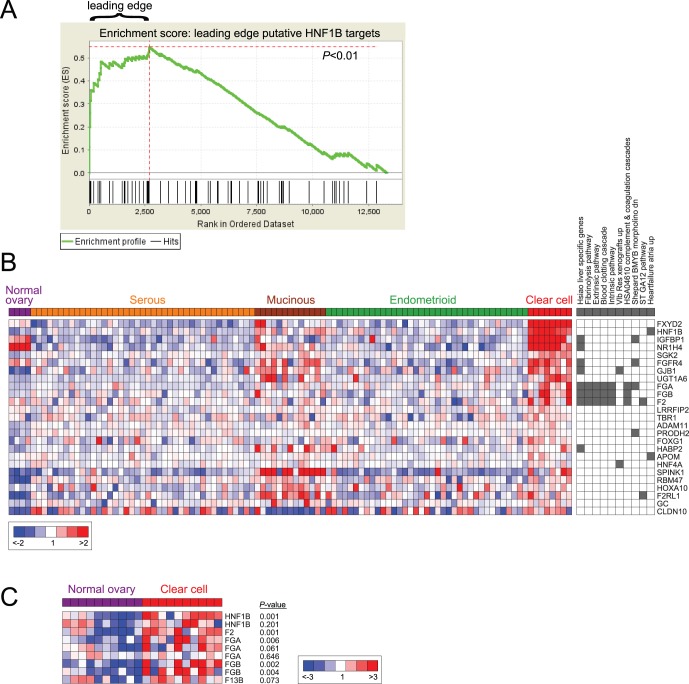
HNF1B transcriptional targets and clotting cascade are enriched in ovarian CCC. (**A**) GSEA analysis shows enrichment of putative HNF1 targets (“leading edge” from Fig. 3) among genes selectively expressed in ovarian carcinoma with clear cell *vs*. other histotype [25]. (**B**) The leading edge enriched genes determined here (n = 25) are significantly over-represented among select functional gene sets (nominal *P* values<0.01); the top 10 gene sets are shown. Columns on the left depict relative log_2_ gene expression levels (red/blue scale indicated) among normal ovary and ovarian carcinomas of different histotype. Columns on the right indicate membership (gray fill) among the top 10 most significant gene sets, which include liver-specific genes, and the clotting cascade (represented by five different gene sets). (**C**) Clotting factor genes (*FGA*, *FGB*, *F2*, and *F13B*) are more highly expressed in laser-microdissected ovarian CCC *vs*. normal ovarian surface epithelium (dataset from [26]). Heatmap depicts all microarray probes for the respective genes; corresponding *P*-values (two-sided Student’s t-test) are indicated.

To investigate the connection between the HNF1B transcriptional program and ovarian CCC, we asked whether the computationally supported HNF1B targets (i.e. the leading edge of putative targets enriched upon HNF1B induction) were selectively expressed in a separate cohort of ovarian CCC. For this analysis, we made use of a publicly-available ovarian cancer microarray dataset [25] that included 8 clear cell, 41 serous, 13 mucinous and 37 endometrioid carcinomas, as well as 4 normal ovary specimens. By GSEA, the set of supported HNF1B targets was significantly enriched among the genes upregulated in ovarian CCC *vs*. other histotypes (*P*<0.01; Fig. 4A). Notably, the leading edge subset (n = 25), i.e. those most associated with clear cell phenotype, retained a significant overlap with the five clotting cascade gene sets (that included genes *FGA*, *FGB* and *F2*) (nominal *P* values<0.01; Fig. 4B). These findings document an HNF1B transcriptional program in ovarian tumors with cytoplasmic clearing, and in particular verify the expression of HNF1B target clotting factor genes in ovarian CCC. Moreover, in an independent microarray dataset [26], clotting factor genes (*FGA*, *FGB*, *F2 and F13B*) were also found more highly expressed in laser microdissected ovarian CCC compared to normal ovarian surface epithelium (Fig. 4C).

**Figure 5:**
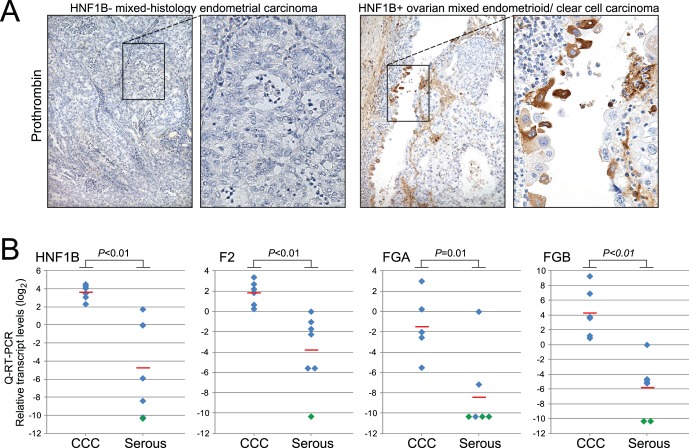
HNF1B-positive gynecologic malignancies are associated with cytoplasmic prothrombin expression, and increased clotting factor transcript levels. (**A**) Representative prothrombin immunostains (10×objective, and magnified inset) are shown for HNF1B-negative, prothrombin-negative mixed-histology endometrial carcinoma (*left*); and HNF1B-positive, prothrombin-positive (moderate cytoplasmic staining) mixed endometrioid/clear cell ovarian carcinoma (*right*; same case as depicted in Fig. 1C). (**B**) Q-RT-PCR analysis demonstrates increased clotting factor transcript levels in ovarian CCC (n = 6) compared to ovarian serous carcinoma (n = 7). Transcript levels are normalized to GAPDH, then set relative to a single serous carcinoma “reference” sample, and reported as log2 values. For graphs shown, undetected transcript levels (green diamonds) are arbitrarily set to smallest detectable levels. Red bars indicate average levels; *P*-values (non-parametric Mann-Whitney U-test) are shown.

To evaluate the relationship between HNF1B and the protein-level expression of clotting factor genes, we performed IHC on 14 gynecologic cancers with cytoplasmic clearing (10 ovarian, 3 endometrial and 1 cervical carcinoma), using an antibody specific to prothrombin [21]. Eight of 10 (80%) HNF1B-positive cases also expressed prothrombin (moderate to strong cytoplasmic staining; Fig. 5A), compared to just 2 of 6 (33%) HNF-negative cases (P = 0.015; two-tailed Fisher’s exact test). Suitable antibodies (IHC-paraffin) could not be identified to assess the protein expression of other HNF1B-associated clotting factors. Nonetheless, Q-RT-PCR analysis corroborated elevated F2, FGA and FGB transcript levels in a small, independent set of freshly-frozen ovarian CCC (n = 6) compared to ovarian serous carcinomas (n = 7) (Fig. 5B; *P*≤0.01, Mann-Whitney U-test).

### HNF1B Expression is Associated with Venous Thromboembolism

The identification of clotting factor genes among HNF1B targets in ovarian CCC suggests the possibility that HNF1B expression in tumors might increase the risk of venous thrombosis. To investigate this hypothesis, we examined a cohort of 38 patients with gynecologic primary tumors (ovarian, endometrium, cervix) that displayed some degree of cytoplasmic clearing (and therefore enriched for HNF1B expression), and for which detailed medical records were accessible (Table S2). Overall, 55% of cases stained positively for HNF1B by IHC, and 37% of patients experienced one or more clinically-significant venous thromboembolic event, i.e. deep venous thrombosis (DVT), pulmonary embolism (PE), or stroke. The relatively high rate of thromboembolic events is commensurate with the reported increased thrombotic risk in ovarian CCC [28], [29]. Notably, patients with HNF1B-positive tumors were significantly more likely (3.0-fold) to experience thromboembolic events (52% for HNF1B-positive cases, *vs*. 18% for HNF1B-negative cases) (*P* = 0.043; two-tailed Fisher’s exact test) (Table 1).

**Table 1:** Tumor HNF1B expression is associated with venous thrombosis.

		No associated venousthrombosis	Associated venousthrombosis	Total	*P*-value^1^
Gynecologic carcinomas	HNF1B-negative	14	3	17	0.043
	HNF1B-positive	10	11	21	
	Total	24	14	38	
Renal carcinomas	HNF1B-negative	40	6	46	0.140
	HNF1B-positive	42	15	57	
	Total	82	21	103	
Combined gynecologic & renal carcinomas	HNF1B-negative	54	9	63	0.011
	HNF1B-positive	52	26	78	
	Total	106	35	141	

1 Two-sided Fisher’s exact test.

We then extended our analysis to a cohort of 103 patients with renal carcinoma (various histologies) for which HNF1 staining had been done and detailed medical records accessible (Table S3). In renal tumors, HNF1B positivity showed a trend towards increased thromboembolic events (*P* = 0.14, two-tailed Fisher’s exact test) (Table 1). Notably, by combining the cohorts of gynecologic and renal neoplasms (n = 141), HNF1B positivity was associated with a strongly significant 2.3-fold increased risk of thromboembolism (*P* = 0.011; two-tailed Fisher’s exact test) (Table 1).

## Discussion

### HNF1B and Cytoplasmic Clearing

The diagnosis of CCC is based on morphologic and histologic features, and can at times be challenging. In our study, we set out to explore the relation between HNF1B expression and cytoplasmic clearing in ovarian cancers, and more broadly in other tumor types. Previously, HNF1B was found to be expressed specifically in ovarian CCC (as compared to other histotypes) [4]–[6], and recently HNF1B immunostaining was reported to be a sensitive (82.5% sensitivity) and specific (95.2% specificity) marker for the diagnosis of ovarian CCC (*vs*. high-grade serous carcinoma) [30]. Here we show that in addition to being expressed in ovarian CCC, HNF1B is in fact expressed in the majority of tumors with cytoplasmic clearing, including renal CCC, endometrial CCC (confirming a recent report [6]), germ cell tumors with yolk sac elements, and ovarian and endometrial carcinomas of mixed histology. This finding has important implications for the evaluation of diagnostically-challenging tumors of the ovary, where HNF1B has been proposed, as part of a diagnostic panel, to distinguish CCC from high-grade serous carcinomas [30]. Specifically, the finding of HNF1B expression in other tumors that commonly enter in the differential diagnosis (renal CCC and yolk sac tumors in particular) warrants caution in the interpretation of this marker in the diagnosis of ovarian tumors, and in the work-up of cancers of unknown primary site.

The strong association between HNF1B expression and cytoplasmic clearing across diverse tumor types supports a possible causal connection. To further explore such a link, we evaluated a set of computationally predicted HNF1B target genes (based on HNF1 binding sites) for enrichment among transcripts upregulated with HNF1B induction in a biologically relevant context (human embryonic kidney cells). Notably, the putative HNF1B targets (i.e. the “leading edge” of the gene set enrichment) showed significant overlap with the annotated “starch & sucrose metabolism” gene set.

Prominent among the overlapping genes is glucose-6-phosphatase (*G6PC*), a key enzyme controlling both gluconeogenesis (glucose generation from metabolic intermediates like lactate) and glycogenolysis (glucose generation from glycogen). Hereditary mutations in *G6PC* are associated with type I glycogen storage disease (von Gierke disease) [31]. It is tempting to speculate that G6PC overexpression and increased gluconeogenesis, perhaps in combination with high glucose uptake (which typifies many tumors), leads to excessive glucose levels favoring glycogen accumulation (evidenced as cytoplasmic clearing). While “starch & sucrose metabolism” did not emerge as a top-ten enriched gene set in ovarian CCC (*vs*. other histotypes), the possible connection between G6PC and cytoplasmic clearing warrants additional investigation.

### HNF1B and Tumor-associated Thrombosis

An entirely unanticipated finding from our study is that HNF1B computationally-supported transcriptional targets, including the subset overexpressed in ovarian CCC (compared to other histotypes), are enriched for genes involved in the blood clotting cascade, in particular fibrinogen (alpha and beta chains), prothrombin, and factor XIII (though F13B was not present in the leading edge for ovarian CCC *vs*. other histotypes). These proteins, mainly produced by the liver, constitute the later stages (i.e. final common pathway) of the clotting cascade [32], where thrombin (activated from prothrombin) converts fibrinogen to fibrin, the main protein component of clots, and factor XIII serves to crosslink fibrin monomers, stabilizing the clot. In gynecologic malignancies with cytoplasmic clearing, the association between HNF1B and prothrombin expression was verified by IHC.

The finding of HNF1B-induced clotting factors led us to hypothesize that patients with tumors expressing HNF1B might be at increased risk for venous thrombosis. Remarkably, in a cohort of patients having gynecologic tumors with some degree of cytoplasmic clearing, those with HNF1B-positive tumors (by IHC) were significantly more likely (3-fold) to experience a clinically-significant thrombotic event (including DVT, PE and thrombotic stroke). The larger, combined cohort of gynecologic and renal neoplasms was also associated with significantly increased (2.3-fold) thromboembolic risk. Clearly, independent confirmatory studies are warranted.

Trousseau, the 19^th^ century French internist, provided the first description of thrombosis as a harbinger of occult malignancy [33]. It is now widely recognized that malignancy is associated with a hypercoagulable state, with protean manifestations ranging from asymptomatic lab values, to superficial thrombophlebitis, venous thromboembolism, thrombotic microangiopathy, disseminated intravascular coagulation, arterial thrombosis and thrombotic endocarditis [34]. About 10% of patients with idiopathic venous thromboembolism have an underlying malignancy detectable with extensive diagnostic investigation [35].

Of hospitalized patients, those with clinically-apparent cancer have higher rates of both initial and recurring thromboembolism [36]. Indeed, thrombosis has been cited as the second leading cause of death in patients with malignancy [37]. Interestingly, the highest rates of venous thrombosis are associated with specific sites and types of cancer, which, though varying by study, often include tumors of the pancreas, ovary, kidney, liver and brain [36], [38], [39]. Of relevance, increased risk of thromboembolism has been reported in ovarian CCC, compared to other ovarian epithelial tumor types [28], [29], where it has been linked to worse survival [29]. Hypercoagulability has also been found in association with renal CCC compared to other histotypes [40].

While widely recognized, however, the pathogenetic mechanisms underlying thrombosis in malignancy remain poorly understood. Virchow, the 19^th^ century German physician, proposed that abnormal clotting resulted from the triad of stasis, vascular injury and a hypercoagulable state [41]. Indeed, thrombosis is influenced by the complex interplay between tumor cells, the hemostatic system, and characteristics of the patient including immobilization, hormonal and chemotherapy, major surgical procedures, as well as minor procedures such as catheter insertion [35], [42]. Proposed prothrombotic mechanisms specific to the tumor cells include the production of mucin, tissue factor (a primary initiator of coagulation), “cancer procoagulant” (a protease activity not yet cloned), cytokines, and adhesion receptors [35], [43].

Our finding that HNF1B induces the heterotypic expression of clotting factors in tumor cells, and in particular distal components of the clotting cascade including prothrombin, fibrinogen and possibly factor XIII, implicates an entirely novel mechanism contributing to the hypercoagulable state in malignancy. Consistent with this possibility, prior studies have found elevated plasma prothrombin [44] and fibrinogen levels [45], [46] associated with increased risk of venous thrombosis. Future studies should clarify whether the expression of clotting factors in ovarian CCC is associated with increased local or systemic plasma levels of clotting factors. Additional investigations should also clarify whether increased clotting factor production characterizes other tumor types that are associated with elevated risk of venous thrombosis. Lastly, while our study provides a series of logical connections implicating a casual role of HNF1B in thromboembolism, proof of causality will require definitive studies, e.g. based on the establishment and characterization of a suitable animal model.

Finally, it has been suggested that in some cancer types the incidence of thromboembolism is sufficiently elevated to warrant prospective clinical trials of primary thromboprophylaxis [47], [48]. In that context, HNF1B may provide a useful biomarker for thromboembolic risk, not only in CCC but also in tumors that do not meet established diagnostic criteria. This latter point is illustrated by two patients in the cohort we studied who had ovarian carcinoma of mixed histology, with associated thrombosis (patients #32 and #33 in Table S2; the tumor from patient #33 is also shown in Figs. 1C and 5B). Though this tumor type has not previously been associated with thrombosis, both tumors expressed HNF1B.

### HNF1B as a Lineage-dependent Cancer Gene Mediating Pleiotropic Phenotypes

Prior studies have linked HNF1B to cancer, though sometimes with contradictory roles. Somatic deletion of *HNF1B* (in combination with germline mutation) has been reported in some chromophobe renal cell carcinomas, suggesting a possible tumor suppressor function [49]. Promoter hypermethylation, silencing HNF1B expression, has also been found in ovarian carcinomas (non-CCC), and in colorectal, gastric and pancreatic cancer cell lines [7]. In contrast, promoter hypomethylation with increased expression has been reported in ovarian CCC [8] (which we have now confirmed and extended to renal CCC), a finding more consistent with an oncogenic role. In support of this, other investigators demonstrated that RNA interference-mediated knockdown of HNF1B in two ovarian CCC cell lines led to apoptotic cell death [4].

This latter finding in particular identifies HNF1B as a possible “lineage-dependent” oncogene [50], an emerging class of oncogenes that are most typically master transcriptional regulators of normal cell lineage that are abnormally expressed in tumors derived from that lineage, where their expression supports tumor proliferation or survival. Other prominent examples include MITF in melanoma [51] and NKX2-1 (TTF1) in lung cancer [52]. However, in contrast with these other genes, *HNF1B* (located on cytoband 17q12) does not appear to be commonly amplified in ovarian CCC [53]. Rather, hypomethylation and expression might reflect a continued expression from the normal cell of origin, or transdifferentiation to a different normal cell type.

In this regard, one might propose that the HNF1B transcriptional program in ovarian CCC reflects the acquisition of a liver-like transcriptional program, as evidenced by the enrichment of liver specific genes, and of the clotting cascade. However, an alternative possibility, and one we favor, is that the HNF1B transcriptional program in ovarian CCC reflects an endometrial secretory cell of origin. While ovarian epithelial carcinomas are generally thought to originate from ovarian surface epithelium, a significant proportion of ovarian CCC is thought to arise from endometriosis (ectopic pelvic endometrium, commonly on the ovaries) [54]. Supportive evidence includes the common association of ovarian CCC (compared to other histotypes) with endometriosis [55], and reports of shared genetic alterations with adjacent endometriosis [56], [57].

Our own findings also support an endometrial origin. For example, prominent among HNF1B target genes enriched in ovarian CCC is *IGFBP1* (insulin growth factor binding protein 1) (Fig. 4), also called placental protein 12. Notably, IGFBP1 is highly expressed in secretory (latter half of the menstrual cycle) and gestational endometrium, where it functions in blastocyst implantation [58], [59]. HNF1B itself has also been reported to be highly expressed in secretory and gestational endometrium [6], and in ovarian endometriosis (atypical or reactive type) associated with CCC [5]. Moreover, glycogen accumulation also characterizes secretory endometrium [60], where it is thought to be essential for nutritional maintenance of blastocyst implantation and early fetal growth. Finally, tightly regulated hemostasis (requiring clotting factors) is critical in normal menses, as well as in implantation and later in delivery [61].

Thus, we speculate that HNF1B-associated cytoplasmic clearing (glycogen accumulation) and clotting factor production in ovarian CCC might reflect the residual, normal functions of an endometrial secretory cell. As such, HNF1B not only contributes to tumor cell survival [4], but elicits pleiotropic phenotypes of clinical significance. In particular, the connection here between HNF1B and clotting factors suggests a novel mechanism underlying tumor-associated thrombosis, with important implications for risk assessment and patient management.

## Conclusions

The hypercoagulable state of malignancy is a well recognized albeit poorly understood phenomenon. Here, by integrative computational analysis we link HNF1B transcription factor expression to the production of blood clotting factors by tumor cells, and to an increased risk of venous thrombosis. Our findings support a novel mechanism of tumor-associated thrombosis in which tumor cells themselves produce clotting factors, and nominate HNF1B as marker for thrombotic risk assessment. These findings have broad implications for understanding malignancy-associated thrombosis, and suggest clear routes to clinical translation, most directly in the potential application of HNF1B as a biomarker for predicting thrombotic risk.

## Supporting Information

Table S1 **Summary of HNF1B immunostaining results.**
(DOC)

Table S2 **Gynecologic carcinomas with cytoplasmic clearing evaluated for HNF1B immunostaining and associated venous thrombosis.**
(DOC)

Table S3 **Renal tumors evaluated for HNF1B immunostaining and associated venous thrombosis.**
(DOC)
