# Supplementary material for: Integrative Bioinformatics Links HNF1B with Clear Cell Carcinoma and Tumor-Associated Thrombosis
Source: PLoS One. 2013 Sep 9;8(9):e74562. doi: 10.1371/journal.pone.0074562 (PMC3767734; doi:10.1371/journal.pone.0074562)
Supplement: Table S1 — Summary of HNF1B immunostaining results. (DOC) [file pone.0074562.s001.doc]

**Table S1. Summary of HNF1B immunostaining results**

| **Category** | **Tumor site** | **Histotype** | **HNF1B+ cases /**  **total cases** |
| --- | --- | --- | --- |
| **Gynecologic neoplasms with cytoplasmic clearing** |  |  | **56/85** |
|  | Endometrial cancer |  |  |
|  |  | Clear cell carcinoma | 4/4 |
|  |  | Secretory carcinoma | 2/2 |
|  |  | Mixed endometrioid and clear cell carcinoma | 3/6 |
|  |  | Mixed serous and clear cell carcinoma | 1/1 |
|  | Ovarian surface epithelial neoplasms |  |  |
|  |  | Clear cell carcinoma | 28/45 |
|  |  | Mixed clear and endometrioid | 9/15 |
|  | Ovarian germ cell tumors |  |  |
|  |  | Yolk sac tumor | 4/6 |
|  |  | Mixed germ cell (yolk sac components) | 2/3 |
|  | Cervical | Clear cell | 3/3 |
|  |  |  |  |
| **Gynecologic neoplasms without cytoplasmic clearing** |  |  | **32/599** |
|  | Ovarian |  |  |
|  |  | Serous carcinoma | 0/216 |
|  |  | Mucinous carcinoma | 0/10 |
|  |  | Endometrioid | 2/32 |
|  |  | Sex cord stromal | 0/14 |
|  |  | Germ cell tumor (other than yolk sac tumor) | 0/4 |
|  | Primary peritoneal |  |  |
|  |  | Serous carcinoma | 0/38 |
|  | Endometrial/ endocervical carcinomas |  |  |
|  |  | Endometrioid | 30/276 |
|  |  | Serous | 0/9 |
|  |  |  |  |
| **Renal clear cell carcinoma, conventional type** |  |  | **166/295** |
|  | Renal |  |  |
|  |  | Clear cell carcinoma, conventional type | 166/295 |
|  |  |  |  |
| **Renal neoplasms without cytoplasmic clearing** |  |  | **19/121** |
|  | Renal |  |  |
|  |  | Chromophobe^1^ | 0/20 |
|  |  | Oncocytoma | 0/16 |
|  |  | Transitional cell carcinoma | 0/45 |
|  |  | Papillary | 19/21 |
|  |  | Angiomyolipoma | 0/19 |
|  |  |  |  |
| **Other neoplasms**^2^ |  |  | **5/393** |

^1^Non-specific cytoplasmic staining noted.

^2^Includes neoplasms of the breast (ductal, lobular, metaplastic); esophagus (squamous, adenocarcinoma, dysplasia); liver (hepatocellular, hemangioendothelioma, hemangioma, cholangiocarcinoma); stomach (adenocarcinoma); pancreas (neuroendocrine, ductal, mucinous cystic neoplasm, serous microcystic adenoma); adrenal (neuroblastomas, carcinoma, pheochromocytoma, adenoma); colon (adenocarcinoma, carcinoid, adenoma); bladder (transitional, adeno, small cell carcinoma); prostate (adenocarcinoma, dysplasia); skin (basal cell, merkel cell, Merkel cell, squamous cell carcinoma, melanoma); bone (osteosarcoma, Ewing’s sarcoma); brain/spinal cord (chordoma, glioma); salivary gland (pleomorphic adenoma, myoepithelial tumor, Warthin tumor, mucoepidermoid, adenoid cystic carcinoma); thymus (thymoma); lung (adenocarcinoma, adenoid cystic, large cell, neuroendocrine, non-small cell, small cell carcinoma, mesothelioma, mucoepidermoid carcinoma, squamous cell carcinoma); thyroid (follicular, medullary, papillary); testis (seminoma); and lymph node (follicular lymphoma, diffuse large B cell lymphoma, chronic lymphocytic leukemia, plasmacytoma).
