# Supplementary material for: Integrative Bioinformatics Links HNF1B with Clear Cell Carcinoma and Tumor-Associated Thrombosis
Source: PLoS One. 2013 Sep 9;8(9):e74562. doi: 10.1371/journal.pone.0074562 (PMC3767734; doi:10.1371/journal.pone.0074562)
Supplement: Table S2 — Gynecologic carcinomas with cytoplasmic clearing evaluated for HNF1B immunostaining and associated venous thrombosis. (DOC) [file pone.0074562.s002.doc]

**Table S2. Gynecologic carcinomas with cytoplasmic clearing evaluated for HNF1B immunostaining and associated venous thrombosis**

| **Case** | **Site** | **Histotype** | **Age** | **HNF1B expression** | **Thromboembolic event**^1^ |
| --- | --- | --- | --- | --- | --- |
| 01 | ovarian | clear cell | 36 | - | - |
| 02 | ovarian | clear cell | 38 | - | - |
| 03 | ovarian | clear cell | 46 | - | - |
| 04 | ovarian | clear cell | 56 | - | - |
| 05 | ovarian | clear cell | 58 | - | - |
| 06 | ovarian | clear cell | 63 | - | - |
| 07 | ovarian | clear cell | 69 | - | - |
| 08 | ovarian | mixed | 61 | - | - |
| 09 | ovarian | mixed | 64 | - | - |
| 10 | ovarian | mixed | 73 | - | - |
| 11 | ovarian | mixed endometrioid | 50 | - | - |
| 12 | ovarian | mixed endometrioid | 88 | - | - |
| 13 | endometrial | mixed | 52 | - | - |
| 14 | endometrial | mixed | 60 | - | - |
| 15 | ovarian | clear cell | 49 | - | + (DVT)^2^ |
| 16 | ovarian | clear cell | 52 | - | + (DVT)^2^ |
| 17 | ovarian | mixed endometrioid | 80 | - | + (stroke) |
| 18 | ovarian | clear cell | 40 | + | - |
| 19 | ovarian | clear cell | 49 | + | - |
| 20 | ovarian | clear cell | 51 | + | - |
| 21 | ovarian | clear cell | 52 | + | - |
| 22 | ovarian | mixed | 64 | + | - |
| 23 | ovarian | mixed endometrioid | 54 | + | - |
| 24 | ovarian | mixed endometrioid | 60 | + | - |
| 25 | ovarian |  | 29 | + | - |
| 26 | ovarian |  | 49 | + | - |
| 27 | ovarian |  | 72 | + | - |
| 28 | ovarian | clear cell | 46 | + | + (DVT)^2^ |
| 29 | ovarian | clear cell | 47 | + | + |
| 30 | ovarian | clear cell | 61 | + | + (DVT) |
| 31 | ovarian | clear cell | 72 | + | + |
| 32 | ovarian | mixed | 68 | + | + (DVT) |
| 33 | ovarian | mixed endometrioid | 46 | + | + (stroke)^2^ |
| 34 | ovarian |  | 39 | + | + (stroke)^2^ |
| 35 | ovarian |  | 51 | + | + (PE) |
| 36 | ovarian |  | 82 | + | + |
| 37 | endometrial | mixed | 60 | + | + |
| 38 | cervical | mixed | 74 | + | + |

^1^From ICD-9 coding and/or other electronic medical records; type of event indicated when known.

^2^Thrombotic event precedent to cancer diagnosis.

Abbreviations: DVT (deep venous thrombosis); PE (pulmonary embolism)
