# Supplementary material for: Integrative Bioinformatics Links HNF1B with Clear Cell Carcinoma and Tumor-Associated Thrombosis
Source: PLoS One. 2013 Sep 9;8(9):e74562. doi: 10.1371/journal.pone.0074562 (PMC3767734; doi:10.1371/journal.pone.0074562)
Supplement: Table S3 — Renal tumors evaluated for HNF1B immunostaining and associated venous thrombosis. (DOC) [file pone.0074562.s003.doc]

**Table S3. Renal tumors evaluated for HNF1B immunostaining and associated venous thrombosis**

| **Case** | **Histotype** | **HNF1B expression** | **Thromboembolic event**^1^ |
| --- | --- | --- | --- |
| 01 | angiomyolipoma | - | - |
| 02 | chromophobe | - | - |
| 03 | chromophobe | - | - |
| 04 | chromophobe | - | - |
| 05 | oncocytoma | - | - |
| 06 | oncocytoma | - | - |
| 07 | papillary | - | - |
| 08 | papillary | - | - |
| 09 | papillary | - | - |
| 10 | papillary & RCC | - | - |
| 11 | RCC | - | - |
| 12 | RCC | - | - |
| 13 | RCC | - | - |
| 14 | RCC | - | - |
| 15 | RCC | - | - |
| 16 | RCC | - | - |
| 17 | RCC | - | - |
| 18 | RCC | - | - |
| 19 | RCC | - | - |
| 20 | RCC | - | - |
| 21 | RCC | - | - |
| 22 | RCC | - | - |
| 23 | RCC | - | - |
| 24 | RCC | - | - |
| 25 | RCC | - | - |
| 26 | RCC | - | - |
| 27 | RCC & angiomyolipoma | - | - |
| 28 | TCC | - | - |
| 29 | TCC | - | - |
| 30 | TCC | - | - |
| 31 | TCC | - | - |
| 32 | TCC | - | - |
| 33 | TCC | - | - |
| 34 | TCC | - | - |
| 35 | TCC | - | - |
| 36 | TCC | - | - |
| 37 | TCC | - | - |
| 38 | TCC | - | - |
| 39 | TCC | - | - |
| 40 | TCC | - | - |
| 41 | chromophobe | - | + (DVT) |
| 42 | RCC | - | + |
| 43 | RCC | - | + |
| 44 | RCC | - | + |
| 45 | TCC | - | + |
| 46 | TCC | - | + |
| 47 | papillary | + | - |
| 48 | papillary | + | - |
| 49 | papillary | + | - |
| 50 | papillary | + | - |
| 51 | papillary | + | - |
| 52 | papillary | + | - |
| 53 | RCC | + | - |
| 54 | RCC | + | - |
| 55 | RCC | + | - |
| 56 | RCC | + | - |
| 57 | RCC | + | - |
| 58 | RCC | + | - |
| 59 | RCC | + | - |
| 60 | RCC | + | - |
| 61 | RCC | + | - |
| 62 | RCC | + | - |
| 63 | RCC | + | - |
| 64 | RCC | + | - |
| 65 | RCC | + | - |
| 66 | RCC | + | - |
| 67 | RCC | + | - |
| 68 | RCC | + | - |
| 69 | RCC | + | - |
| 70 | RCC | + | - |
| 71 | RCC | + | - |
| 72 | RCC | + | - |
| 73 | RCC | + | - |
| 74 | RCC | + | - |
| 75 | RCC | + | - |
| 76 | RCC | + | - |
| 77 | RCC | + | - |
| 78 | RCC | + | - |
| 79 | RCC | + | - |
| 80 | RCC | + | - |
| 81 | RCC | + | - |
| 82 | RCC | + | - |
| 83 | RCC | + | - |
| 84 | RCC | + | - |
| 85 | RCC | + | - |
| 86 | RCC | + | - |
| 87 | RCC | + | - |
| 88 | RCC | + | - |
| 89 | papillary | + | + |
| 90 | papillary | + | + |
| 91 | papillary | + | + |
| 92 | papillary | + | + (stroke)^2^ |
| 93 | papillary | + | + |
| 94 | RCC | + | + |
| 95 | RCC | + | + |
| 96 | RCC | + | + |
| 97 | RCC | + | + |
| 98 | RCC | + | + (DVT)^2^ |
| 99 | RCC | + | + |
| 100 | RCC | + | + |
| 101 | RCC | + | + |
| 102 | RCC | + | + (DVT)^2^ |
| 103 | RCC | + | + (DVT)^2^ |

^1^From ICD-9 coding and/or other electronic medical records; type of event indicated when known.

^2^Thrombotic event precedent to cancer diagnosis.

Abbreviations: DVT (deep venous thrombosis); PE (pulmonary embolism); RCC (renal cell carcinoma); TCC (transitional cell carcinoma)
